# Application of a “nursing education cloud platform”-based combined and phased training model in the education of standardized-training nurses: A quasi-experimental study

**DOI:** 10.1097/MD.0000000000044138

**Published:** 2025-08-22

**Authors:** Haoyu Tang, Yuan Yuan, Huaxi Liu, Shaoyi Hu

**Affiliations:** a Department of Nursing, The First Affiliated Hospital of Army Medical University, Chongqing, China; b Department of General Medicine, The First Affiliated Hospital of Army Medical University, Chongqing, China; c Department of Preventive Medicine, The First Affiliated Hospital of Army Medical University, Chongqing, China.

**Keywords:** clinical teaching, nursing education cloud platform, service learning, standardized-training nurses, training model

## Abstract

The evolution of nursing education has rendered traditional standardized-training models increasingly inadequate, primarily due to their inflexible curricula, limited personalized instruction, and delayed feedback loops. While stage-based training models offer improved coherence through structured planning, they encounter difficulties in resource integration and real-time interaction. Contemporary advancements in cloud computing and Internet of Things technologies present novel opportunities for educational reform. Nursing Education Cloud Platform (NECP)-based systems have demonstrated efficacy in medical education, particularly in efficient resource management, data-driven decision-making, and the design of adaptable learning pathways. Despite the nascent implementation of cloud platforms in standardized nurse training, the sustained impact on multifaceted competencies, including professional identity and clinical reasoning, warrants further investigation. The primary objective of this investigation was to assess the effectiveness of a NECP-integrated, phased training model in enhancing standardized-training nurses’ theoretical comprehension, practical competencies, professional self-perception, and clinical decision-making capabilities, while also examining its potential to refine nursing education methodologies. This quasi-experimental, non-randomized controlled trial evaluated the impact of a NECP-based training program. The study encompassed an experimental group (n = 56, receiving cloud platform-based training from September 2021 to August 2022) and a control group (n = 56, undergoing traditional training from September 2020 to August 2021). Group assignment was determined by the hospital’s annual training schedule, thus employing a natural grouping based on the time period. Propensity score matching was utilized to mitigate baseline characteristic imbalances. The intervention’s effects were assessed across several domains, including theoretical knowledge, operational skills, professional identity, and clinical reasoning abilities. ANCOVA was employed to account for temporal covariates. The experimental group scored significantly higher than the control group in theoretical knowledge (88.70 ± 5.07 vs 75.55 ± 9.01, *P* < .05), operational skills (94.27 ± 2.04 vs 90.95 ± 3.69, *P* < .05), professional identity (73.18 ± 10.18 vs 62.54 ± 15.48, *P* < .05), and clinical reasoning ability (60.95 ± 8.90 vs 51.09 ± 12.28, *P* < .05). The integration of the “NECP” with a phased training model demonstrates efficacy in augmenting nurses’ competencies. However, the potential for selection bias, inherent in the non-randomized design, warrants careful consideration in the interpretation of these findings. Further investigation, specifically through multicenter longitudinal studies, is recommended to ascertain the generalizability of these results.

## 1. Introduction

With the rapid development of nursing education, traditional standardized-training models have increasingly revealed multiple limitations. Firstly, their fixed curricula struggle to accommodate individualized learning needs, leading to inconsistent training outcomes.^[1]^ Following graduation, nursing students transition directly into clinical practice, encountering challenges related to identity formation and professional competency. Their existing knowledge base, clinical reasoning abilities, and skill proficiency often prove inadequate relative to the demands of their clinical roles.^[2]^ Secondly, standardized training for nurses constitutes a critical phase preceding independent clinical practice, thereby facilitating adaptation to the clinical environment and the acquisition of essential competencies. This standardized-training process is of paramount importance in fostering the professional development of nurses.^[3,4]^ In recent years, network information platforms have been progressively integrated into training as educational tools. The proliferation of the “nursing education cloud platform (NECP)” and portable digital devices has reshaped the pedagogical landscape. Their utility lies in circumventing temporal and spatial constraints, thereby facilitating nurses’ systematic assimilation of professional knowledge throughout their learning trajectory, and significantly augmenting higher education, including nursing education.^[5,6]^ Furthermore, phased training can transform abstract theoretical knowledge from rote memorization to comprehension, subsequently facilitating its application within clinical practice, thereby rendering training objectives more explicit and focused.^[7]^ Within the extant standardized nursing education paradigm, the conventional training model presents several limitations, including a rigid curriculum, a deficiency in personalized mentorship, and challenges in the objective quantification of training effectiveness.^[8]^ While the phased training model has, to a certain degree, enhanced the systematic approach to training, it continues to demonstrate deficiencies in resource integration and real-time feedback.^[9]^ Furthermore, extant research primarily emphasizes the examination of a singular training paradigm, with a deficiency in the comprehensive assessment of the integration of the NECP within the phased training model. Consequently, this investigation endeavors to ascertain the application efficacy of the NECP-based phased training model in the education of standardized-training nurses. This methodology seeks to mitigate the constraints of conventional training models, augment the personalization and scientific rigor of training, and furnish practical references for the domain of nursing education.The NECP represents an online education management system predicated on cloud computing infrastructure. Its primary functions include resource aggregation, data analytics, real-time feedback loops, and the formulation of personalized learning pathways. Through a modular architecture, encompassing account administration, online assessment tools, and learning progress monitoring, the platform facilitates comprehensive management of standardized-training protocols. Moreover, it overcomes the temporal and spatial constraints inherent in conventional training methodologies, thereby optimizing instructional efficacy.^[10]^ By comparing and analyzing the application effects of the phased training model based on the NECP and the traditional training model in the teaching of standardized-training nurses, the effectiveness and feasibility of this model will be further verified.^[11]^ At the same time, this study will explore the specific roles of the NECP in improving nurses’ theoretical knowledge, operational skills, professional identity, and clinical reasoning abilities, to provide novel insights and methodologies for advancing the innovation and development of nursing education.

## 2. Materials and methods

### 2.1. Research subjects

This quasi-experimental, non-randomized controlled trial included nurses who underwent standardized training at a tertiary hospital in Chongqing, China, from September 2020 to August 2022. A sample size calculation indicated a target enrollment of 120 nurses. Group assignment was determined by the hospital’s annual training schedule, resulting in a natural grouping: a control group (September 2020 to August 2021, n = 60) and an experimental group (September 2021 to August 2022, n = 60), without randomization. The attrition rate was 6.67% (8/120), yielding a final analyzable sample of 112 nurses (control n = 56, experimental n = 56). No significant baseline differences were detected between groups (*P* > .05). Propensity score matching (PSM) was utilized to mitigate baseline differences in age, gender, educational background, and pre-training theoretical scores. Intervention effects were evaluated by assessing pre- and post-intervention changes in theoretical knowledge, operational skills, professional identity, and clinical reasoning ability.

Inclusion criteria encompassed: Newly onboarded nurses undergoing standardized training; Possession of a valid nursing professional qualification certificate; Provision of informed consent for participation in this study and commitment to the complete training program.

Exclusion criteria: individuals unable to complete the entire training program due to specific circumstances during the training period; those who withdrew mid-study; those with mental, psychological, or emotional disorders. For participants who withdrew mid-study, the reasons for withdrawal were documented, and corresponding samples were supplemented to maintain equal sample sizes between the 2 groups. No statistically significant differences were observed in gender, age, and educational background between the 2 groups of standardized-training nurses (*P* > .05) (Table 1).

**Table 1:** Comparison of general data of standardized-training nurses in the 2 groups [x ± s/case (%)].

Group	Gender		Age, ($\bar{x}\pm \;s$)	Educational background	
	Male	Female		Junior College	Bachelor
Experimental group(n = 56)	5	51	22.70 ± 1.49	43	13
Control group(n = 56)	3	53	22.27 ± 1.36	42	14
*t*/χ2	0.538		−1.593	0.049	
*P*	.716		.114	.825	

Table 1 delineates the demographic profiles of nurses in the experimental and control cohorts, encompassing gender, age, and educational attainment. No statistically significant differences were detected between groups regarding gender distribution (male: 5 vs 3; female: 51 vs 53) or educational qualifications (bachelor’s degree: 13 vs 14; associate degree: 43 vs 42). Mean ages were comparable (experimental group: 22.70 ± 1.49; control group: 22.27 ± 1.36), with inferential statistics confirming no significant age disparity (*P* = .114). These findings confirm baseline equivalence, supporting group comparability.

### 2.2. Methods

#### 2.2.1. Ethical considerations

This investigation was conducted in accordance with the Declaration of Helsinki and was reviewed by the Ethics Committee of the First Affiliated Hospital of Army Medical University. Considering that the educational interventions did not necessitate the collection of patient-identifiable data or the provision of clinical treatment, the Ethics Committee waived the requirement for a comprehensive ethical review and granted an exemption (without an assigned approval number). However, in the administration of the questionnaire, participants were explicitly informed of the study’s objectives, their right to voluntary participation, the duration of the questionnaire, the guarantee of anonymity, the option to withdraw at any time, and the data privacy and security protocols, in alignment with the principle of informed consent. Prior to survey commencement, participants were required to confirm 4 statements to ensure informed consent: “I understand the purpose of this study and what is expected of me as a participant”; “I acknowledge that my participation is voluntary and that I may withdraw from the study at any time without penalty”; “I am aware that my responses will be kept anonymous and that my personal data will be kept confidential and will not be shared with third parties”; and “I consent to participate in this study and provide my informed consent by proceeding with the survey.”

#### 2.2.2. Sample size calculation

Based on the effect size from prior educational intervention studies (Cohen d = 0.8, α = 0.05, β = 0.2, power = 80%), the sample size was estimated using G*Power 3.1 for independent t-tests. The calculation indicated a minimum requirement of 47 participants per group to detect significant between-group differences. Accounting for an anticipated attrition rate of 20%, we enrolled 60 nurses per group (total N = 120) to ensure adequate statistical power. The actual attrition rate was 6.67% (8/120), resulting in a final analyzable sample of 100 nurses (control n = 56, experimental n = 56), which still met the minimum sample size criteria.

#### 2.2.3. Group allocation:

The allocation of participants to experimental and control conditions was contingent upon the hospital’s established training program, which inherently stratified participants by temporal cohort. Trainees from the 2020 to 2021 cohort received conventional training, whereas those from the 2021 to 2022 cohort were exposed to an intervention delivered through a cloud-based platform. Despite the absence of random assignment, we implemented the following methodologies to mitigate confounding variables:

PSM: covariates including age, gender, educational attainment, and pre-training theoretical scores were integrated. Nearest neighbor matching, employing a caliper value of 0.1, was utilized, thereby achieving balance in baseline characteristics between groups (standardized mean difference < 0.1).

Temporal covariate adjustment: In the statistical analyses, the training year was incorporated as a covariate to account for the influence of temporal factors, such as policy modifications and faculty adjustments.

#### 2.2.4. Control group

The conventional training methodology was implemented: preemployment Training: theoretical instruction was delivered offline from the 1st to the 5th day. The curriculum primarily comprised foundational nursing principles, specialized nursing knowledge, hospital protocols, and medical statutes, among other topics. From the 6th to the 10th day, practical skills training was conducted through demonstrations of operational techniques and group-based feedback. The training regimen included 7 clinical professional operational procedures, including cardiopulmonary resuscitation, intramuscular injections, and intradermal injections. Clinical rotation: subsequent to the assessment syllabus for standardized-training nurses, as formulated by the Chongqing Municipal Health Commission,^[12]^ a structured rotation plan was implemented. Standardized-training nurses were required to rotate through internal medicine, surgery, and selected specialties (emergency medicine department, intensive care unit, operating room). The rotation cycle for each department was 3 months. During the department-learning period, trainees participated in 2 nursing teaching ward rounds and 2 special lectures every month, and 1 difficult-case discussion every quarter. Completion assessment: following 1 year of training, the Nursing Department administered assessments of theoretical knowledge and operational skills to all standardized-training nurses and distributed questionnaires on professional identity and clinical reasoning ability to evaluate the multifaceted skill development of nurses. during the standardized-training period, including the recognition of professional values, professional sense of belonging, and the ability to analyze problems, formulate nursing plans, and implement interventions in clinical practice.While the traditional training model also incorporated phased training (prejob training, clinical rotation, and completion assessment), its phased content was relatively fixed, lacking personalized guidance and a real-time feedback mechanism.In contrast, the innovative phased training model, on the basis of the traditional one, combined with the NECP for dynamic management.This approach enabled the identification of trainees’ weaknesses through data analysis, the provision of personalized learning resources and real-time feedback, and the enhancement of training specificity and flexibility.

#### 2.2.5. Experimental group

**Figure 1.**
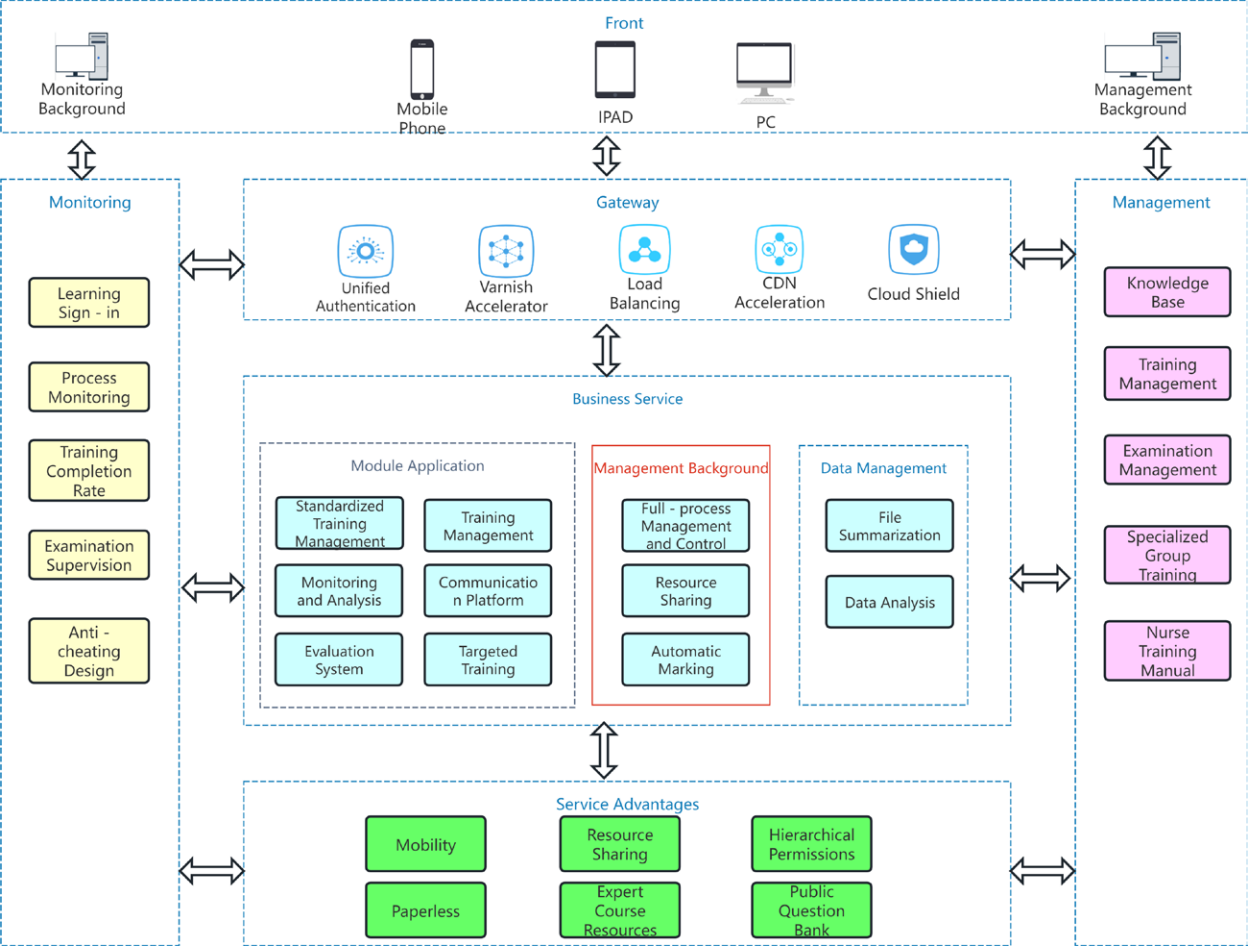
Depicts the comprehensive operational framework of the cloud platform, encompassing the infrastructure layer, platform service layer, and application functional modules. The infrastructure layer comprises computational resources, storage nodes, and network configurations, underpinning the platform’s core functionalities. The platform service layer integrates data management, security authentication, and API interfaces to facilitate dynamic resource allocation and multi-terminal collaboration. The application layer includes modules such as training administration, real-time monitoring, and data analytics. The architectural design prioritizes modularity and scalability, ensuring robust and reliable technical support for phased training programs. API = application programming interface.

**Figure 2.**
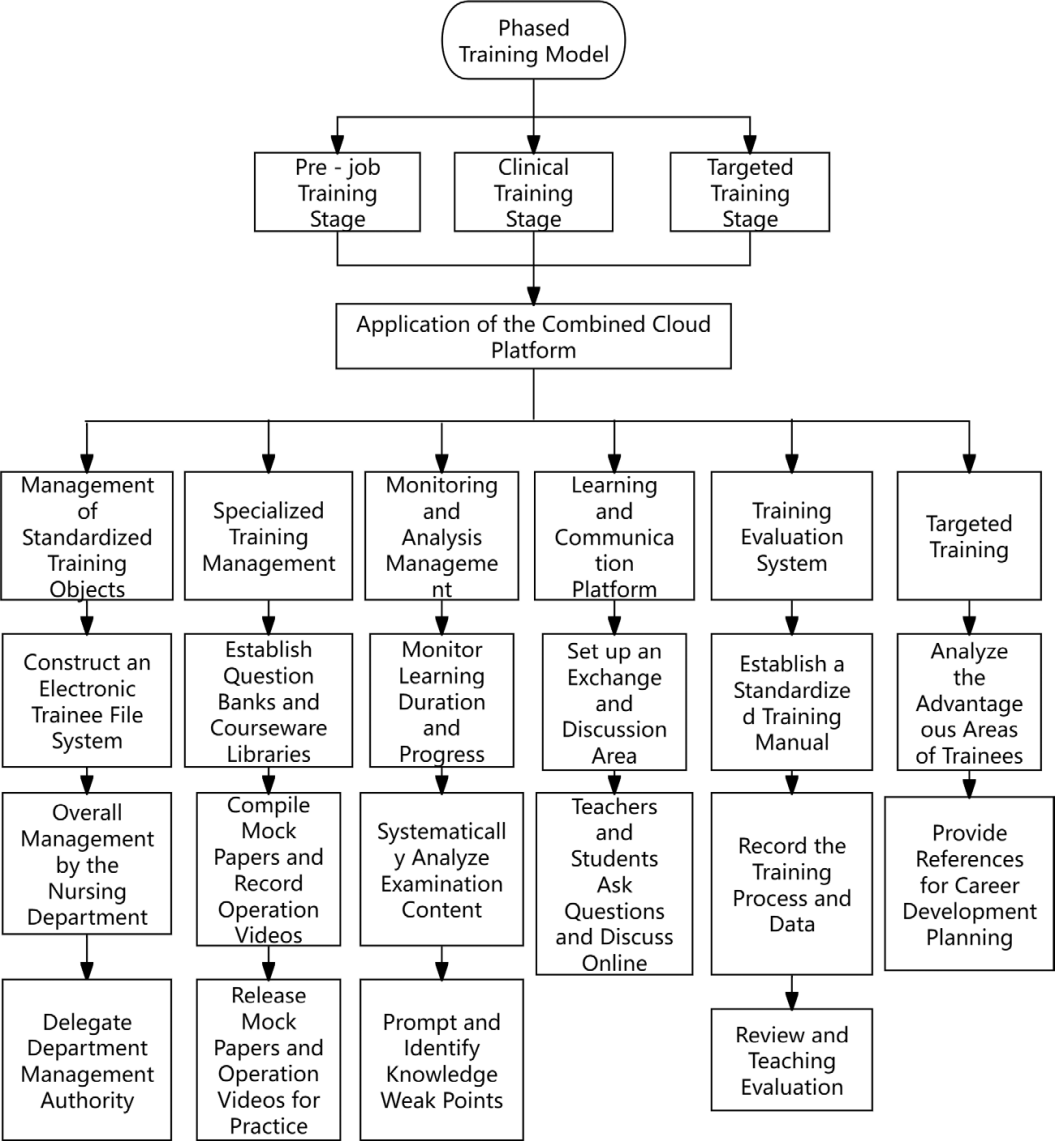
Illustrates a detailed implementation framework for joint phased training leveraging the cloud platform. The training is segmented into 3 phases: preemployment training (online coursework and professional ethics), clinical rotation (clinical reasoning enhancement and remote collaborative exercises), and targeted training (personalized learning pathways and career development). Each phase is seamlessly integrated via the cloud platform, enabling individualized learning trajectories and progress monitoring, thereby ensuring the effective integration of theoretical knowledge and practical application, and facilitating systematic, intelligent management of standardized nurse training.

On the basis of the training of the control group, the NECP (Fig. 1) was used in combination with the phased training model (Fig. 2).

1. Functions of the NECP.The NECP (version:V3.1.6) is designed for online education and career development within the nursing sector, offering structured, professional training services for nurses and nursing practitioners. The NECP system was engineered with Java, employing a browser/server (B/S) architecture to ensure multi-terminal accessibility across personal computers and mobile devices. The backend infrastructure incorporates a MySQL database and is built upon the Spring Boot framework. Interfacing with the Hospital Information System is achieved through standardized application programming interface (API) protocols, facilitating real-time training data synchronization. Server deployment on Alibaba Cloud is implemented to accommodate high-concurrency access, with secure data transmission secured via secure sockets laye/ transport layer security (SSL/TLS) protocol. Security protocols include a web application firewall for attack mitigation, token-based authentication, form parameter validation, and role-based access control, thereby ensuring adherence to medical information security standards.

Its core functionalities encompass: account management module: this module facilitates the creation of administrator and individual user accounts, differentiated by level, department, user role, and position. The user management list module is employed for entering essential personnel data, including gender, name, age, department, type, and other informations. Training and learning module: this module encompasses educational and training plans, including specialized training, orientation for new-nurse training, professional question banks, and technical procedure scoring sheets. It provides video content such as expert lectures and the latest industry updates, and supports teaching courseware and question banks in various formats.A plan management feature allows for the modification creation, export, or deletion of training plans for efficient querying and operation. Online examination module: theoretical assessments are presented electronically, with configurable parameters including scores, examination duration, and the number of answer attempts. During the examination, page navigation is restricted, and upon completion, results are automatically uploaded to the central database.Administrators can access assessment outcomes via a dedicated management interface. Performance statistics and feedback module: each assessment result automatically generates a report card, and supports export in Excel format. It can statistically analyze data such as the pass rate and average score of each department. A question set for incorrect responses is available in the personal center to assist nurses identifying knowledge deficiencies and facilitate remediation. Terminal device module: this module supports online learning and training on terminal devices including mobile applications and PC terminals.The mobile-end applications provides functions such as activity news notifications, activity names, sign-in, and questionnaires. The questionnaires are used to evaluate the satisfaction with the online training content and the training effectiveness.

(2) Application of the NECP modules: Standardized-Training Management: An electronic trainee file system was established for newly admitted standardized-training nurses, with department management permissions configured to ensure the effective implementation of in-department training management. Specialized training management: based on the assessment syllabus for standardized-training nurses formulated by the Chongqing Municipal Health Commission and guided by clinical nursing experts, 30 sets of mock test papers were compiled and released, and operation-standard videos were recorded to build a courseware library.The video courses which include 7 technical operations: cardiopulmonary resuscitation, intramuscular injection, intradermal injection, aseptic technique, intravenous infusion, sputum suction, and oxygen inhalation.These are updated monthly on the NECP for independent trainee study. Monitoring and analysis management: monitor the duration and progress of trainee education and learning, analyze the examination content, identify knowledge gaps, and implement early-warning prompts for trainees who have not completed the required tasks, thereby improving training efficacy. Learning and communication platform: an exchange and discussion area was established to encourage trainees to actively ask questions and engage in discussions. The teacher staff provides timely feedback to jointly promote the continuous improvement of teaching quality. Training evaluation system: a standardized-training manual was established to systematically record the training process of trainees. Trainees update the completion of department teaching tasks every month, and the head nurse conducts strict review and teaching evaluation to ensure the objectivity and fairness of the evaluation results. Targeted training management: based on a comprehensive analysis of trainees’ specialized-learning situations, assessment results, and data in the standardized-training manual, the system analyzes the advantageous areas of each trainee and provides targeted reference opinions for the career development planning of trainees.

(3) The phased training model comprises following: prejob training stage: from first day to tenth day, centralized training is conducted, encompassing professional knowledge, scientific research training, and career planning, etc. Nursing experts including Nightingale Medal recipients and senior professors are invited to deliver lectures, sharing insights on personal growth trajectories, team support and collaboration, etc, thereby, providing valuable references for the career planning of standardized-training nurses. Upon completion of the training, standardized-training nurses are required to complete practice questions related to the prejob training content on the NECP to reinforce learning outcomes. Clinical training stage: a structured rotation plan is implemented from the first to the 8th month, in accordance with the assessment syllabus of the Chongqing Municipal Health Commission, a structured rotation plan is implemented. Standardized-training nurses complete clinical knowledge practice questions related to teaching ward rounds and case discussions on the NECP to cultivate clinical thinking and reasoning abilities. Standardized-training nurses raise specialized nursing questions, and through literature retrieval and the guidance of teaching teachers, they formulate solutions and conduct exchanges and discussions on the NECP to promote clinical application. Standardized-training nurses complete teaching tasks and clock-in on the NECP according to the specialized system they are in, including no <10 class hours of management training, no <6 class hours of specialized-system training, and no <13 class hours of department training. The system will issue early-warnings to trainees who do not meet the standards every month, and through continuous tracking and quantitative analysis, it helps departments to keep abreast of trainees’ learning situations in real-time and adjust teaching strategies promptly. Targeted training stage: from the 10th to the 12th month, integrating data from the NECP, departmental evaluations, and individual preferences, a customized training trajectory is established for each standardized-training nurse to further refine the training path.They can select from 5 major domains: education and training, scientific research and evidence-based practice, professional etiquette, chronic disease management, and intensive care. Standardized-training nurses then undergo a 3-month fixed-department rotation. The teaching department assists them in determining the sub specialty training direction based on prior performance and personal interests, thereby deepening learning in specific fields and dynamically adjusting the learning plan. Standardized-training nurses solve relevant nursing problems through evidence-based reasoning and disseminate their findings across the department to demonstrate learning outcomes. Simultaneously, the shared content is disseminated on the NECP for all trainees to study and discuss, fostering knowledge sharing and the establishment of a team-learning environment. Following the 1 year training period, the Nursing Department conducts theoretical and operational assessments for all standardized-training nurses and distributes questionnaires assessing professional identity and clinical reasoning abilities.

### 2.3. Assessment and evaluation indicators

#### 2.3.1. Theoretical examination scores

Assessments were administered before the commencement of training and upon its completion (the 12th month). An online examination platform was utilized on the NECP. The examination consisted of 100 questions, including 90 single-choice and 10 multiple-choice questions. The examination content was based on the assessment syllabus for standardized-training nurses upon department exit, encompassing fundamental nursing, internal medicine nursing, surgical nursing, specialized nursing, and related topics.

#### 2.3.2. Operational skills scores

Assessments were conducted prior to the commencement of training and upon its conclusion (month 12). Employing the 3 – station assessment protocol established by the National Health Commission of Chongqing Municipality,^[13]^ the first station evaluated fundamental emergency skills specifically cardiopulmonary resuscitation; the second station assessed injection techniques with intramuscular and intradermal injections as evaluation items (participants selected one); the third station encompassed comprehensive skills including aseptic technique, intravenous infusion, sputum suction, and oxygen inhalation (participants selected 1 of the 4). Each station was graded out of 100 points, and the final operational skills score represented the mean of the scores from the 3 skills assessments.

#### 2.3.3. Professional identity

Questionnaires were distributed before and after the training. Respectively, the professional identity scale for nursing students. This scale scale encompasses 5 dimensions: professional self-concept, benefits of job retention and risks of job departure, social comparison and self-reflection, autonomy in career choice, and social persuasion, with a total of 17 items. The scale adopts the Likert-5-level scoring method, and the total score ranges from 17 to 85 points.A higher the score indicates a stronger the professional identity.

#### 2.3.4. Clinical reasoning ability

Questionnaires were distributed respectively before and after the training. The self-rated nurse clinical reasoning ability scale revised by Wang Yujing^[14]^ was utilized, comprising a total of 15 items and employing a Likert-5-level scoring method: in the importance evaluation, scores of 1 to 5 points represented very unimportant, unimportant, moderately important, important, and very important, respectively, in the execution frequency assessment, scores of 1 to 5 points represented never, relatively seldom, average, relatively often, and very often, respectively. The total score ranges from 15 to 75 points, with higher score indicating stronger reasoning ability. Members of the research team distributed electronic questionnaires to standardized-training nurses through the online platform before and after the training. A unified guiding instruction was provided on the first page of the questionnaire, detailing the purpose, significance, and completion method of the survey. Respondents completed the the questionnaire anonymously, adhering to the principle of informed consent, and all questions were designated as required items.

### 2.4. Statistical treatment

SPSS 26.0 (Chicago) statistical software was employed for data analysis and processing. Enumeration data were expressed as the number of cases or percentages, and the χ^2^ test was utilized for inter group comparisons. Measurement data were described using mean and standard deviation, and the t-test was used for inter group comparisons. A and *P*<.05 was considered statistically significant.

### 2.5. Missing data handling

Missing data for all variables were systematically documented. The number and proportion of missing values in the experimental group (n = 56) and control group (n = 56) (Table 2). Missing data primarily resulted from incomplete questionnaires or unassessed evaluations. Multiple imputation was employed to handle missing values, with the imputation model incorporating all analytical variables and baseline characteristics. Sensitivity analyses confirmed that the missing data handling method did not significantly alter the primary outcomes.

**Table 2:** The quantity and proportion of missing values within the experimental and control groups.

Variable	Experimental group missing (proportion)	Control group missing (proportion)
Age	0 (0%)	0 (0%)
Gender	1 (1.79%)	0 (0%)
Educational background	0 (0%)	1 (1.79%)
Pre-training scores	2 (3.57%)	1 (1.79%)
Theoretical exam scores	0 (0%)	0 (0%)
Operational skills	1 (1.79%)	1 (1.79%)
Professional identity scale	3 (5.36%)	2 (3.57%)
Clinical reasoning scale	2 (3.57%)	1 (1.79%)

Table 2 details the rates of missing data for primary variables across experimental and control groups. The experimental cohort demonstrated minimal missingness in gender, operational skills, and professional identity measures (1.79%–5.36%), whereas the control cohort exhibited low missing data rates in educational background, operational skills, and clinical reasoning assessments (0%–3.57%). Missing data proportions remained below 5.36% in both groups, indicating robust data integrity with negligible influence on subsequent statistical analyses.

## 3. Results

### 3.1. Comparison of the theoretical knowledge and operational skills levels of standardized-training nurses in the 2 groups

There was no significant difference in the theoretical knowledge and operational skills levels of the control group before and after the training (*P* > .05), indicating that the traditional training model has limited effects on improving the abilities of standardized-training nurses in the short term. This may be related to the fixed nature of the traditional training model and the lack of personalized guidance.However, the experimental group, through the phased training model combined with the NECP, significantly improved the theoretical knowledge and operational skills levels (*P* < .05), suggesting that the innovative training model has significant advantages in enhancing the abilities of standardized-training nurses (Table 3).

**Table 3:** Comparison of the theoretical knowledge levels and operational skills levels of standardized-training nurses in the 2 groups before and after training [x ± s/case (%)].

Group	Theoretical knowledge level		Operational skills level	
	Before Training	After training	Before training	After training
Experimental group (n = 56)	72.05 ± 9.72	88.70 ± 5.07	89.35 ± 3.57	94.27 ± 2.04
Control group (n = 56)	71.34 ± 7.14	75.55 ± 9.01	89.91 ± 3.63	90.95 ± 3.69
*t* value	−0.443	−9.519	1.621	−5.895
*P*-value	.069	.002	.564	.004

The scores before training are the scores when participating in the prejob training.

Table 3 presents a comparative analysis of theoretical knowledge and practical skill proficiency among nurses in both cohorts pre- and post-intervention. The experimental group exhibited a statistically significant increase in theoretical knowledge scores following training (72.05→88.70, *P* = .002) alongside a notable improvement in practical skills (89.35→94.27, *P* = .004). The control group demonstrated more modest gains (theoretical: 71.34→75.55; practical: 89.91→90.95), with significant differences observed (theoretical *P* = .002, practical *P* = .004). These findings suggest superior training effectiveness within the experimental cohort.

### 3.2. Comparison of the professional identities of standardized-training nurses in the 2 groups

Prior to the intervention, no significant differences were observed in the total scores or the dimensional scores between the standardized-training nurses in the 2 groups (*P* > .05). Following the intervention, significant differences were noted in the total scores and the dimensional scores between the standardized-training nurses in the 2 groups (*P* < .05) (Table 4).

**Table 4:** Comparison of the professional identities of standardized-training nurses in the 2 groups [x ± s/case (%)].

Group	Professional self – concept		Benefits of staying in the job and risks of leaving the job		Social comparison and self-reflection		Autonomy in career choice		Social persuasion		Total score	
	Before training	After training	Before training	After training	Before training	After training	Before training	After training	Before training	After training	Before training	After training
Experimental group (n = 56)	16.04 ± 5.46	25.63 ± 4.13	11.75 ± 4.22	16.63 ± 2.90	9.27 ± 3.80	13.23 ± 2.12	5.98 ± 2.39	8.71 ± 1.52	5.50 ± 2.59	8.98 ± 1.34	48.54 ± 9.54	73.18 ± 10.18
Control group (n = 56)	13.14 ± 5.59	22.14 ± 6.06	10.91 ± 4.03	13.75 ± 4.10	8.04 ± 3.20	10.55 ± 3.10	4.52 ± 1.87	6.29 ± 2.29	5.82 ± 2.82	7.77 ± 2.28	42.43 ± 9.99	62.54 ± 15.48
*t* value	−2.770	−3.552	−1.077	−4.286	−1.855	−5.337	−3.609	−6.601	0.628	−3.435	−3.310	−4.299
*P*-value	.729	.007	.507	.013	.167	.019	.141	.002	.422	.001	.914	.023

Table 4 evaluates multiple facets of professional identity across the 2 nurse groups. The experimental group showed significant post-training enhancements in the dimensions of “professional self-concept,” “retention benefits,” and “social comparison and reflection” (all *P* < .05), with total scores rising from 48.54 to 73.18 (*P* = .023). The control group exhibited a smaller increase in total scores (42.43→62.54), with statistical significance detected only in the “retention benefits” dimension (*P* = .013). Collectively, the experimental group demonstrated greater advancement in professional identity metrics.

### 3.3. Comparison of the clinical reasoning abilities of standardized-training nurses in the 2 groups

Prior to the intervention, the clinical reasoning capabilities of standardized-training nurses in both cohorts were comparatively limited, with no statistically significant intergroup disparity (*P* > .05). Post-intervention, the clinical reasoning abilities improved in both groups increased, however, the experimental group demonstrated a significantly greater increase than the control group, and this difference reached statistical significance (*P* < .05) (Table 5).

**Table 5:** Comparison of the clinical reasoning abilities of standardized-training nurses in the 2 groups (n = 112).

Group	Before training	After training
Experimental group(n = 56)	43.14 ± 14.53	60.95 ± 8.90
Control group(n = 56)	40.21 ± 14.13	51.09 ± 12.28
*t* value	−1.082	−4.863
*P*-value	.824	.014

Table 5 compares clinical reasoning competencies between the 2 nurse cohorts. The experimental group’s clinical reasoning scores significantly improved post-training (43.14→60.95, *P* = .014), whereas the control group showed a less pronounced increase (40.21→51.09, *P* = .014). Intergroup post-training differences were statistically significant (T = −4.863), indicating that the intervention yielded a more substantial enhancement in clinical reasoning within the experimental group.

## 4. Discussion

The NECP developed in this study integrates diverse educational resources and technical tools, providing standardized-training nurses with an efficient and flexible learning and development planning platform.In recent years, higher education institutions have accelerated the exploration of remote online teaching models for nursing majors. Through small-scale restricted online classrooms such as the China University Massive Open Online Course (MOOC),^[15]^ WeChat Rain Classroom,^[16]^ and Chaoxing Learning Tong Platform,^[17]^ the process of remote and digital nursing education has been further promoted. Information-based management has demonstrated significant superiority in aspects such as data integration, real-time monitoring, and decision-making assistance in standardized-training management.^[18]^ The “online + offline” hybrid training model has effectively enhanced the flexibility of teaching resources, thus significantly promoting the enthusiasm and compliance of nurses in learning.^[19]^ Through the application of the information-based management platform, the flat-teaching management mode implemented by the Nursing Department and the teaching backbones of each depa.rtment has been transformed into a more efficient management structure.^[20]^

### 4.1. The promotion of theoretical knowledge and operational skills by the NECP

Standardized-training, traditionally implemented within individual departments, exhibits variable efficacy.^[21]^ This variance stems from disparities in training methodologies, the relevance of the material, and the instructors’ qualifications. The NECP addresses these limitations by offering structured learning resources for standardized-training of nurses, integrating 30 sets of mock examinations, 7 operational standard videos, and a dynamically updated courseware repository. This approach enhances the training process’s accessibility and effectiveness.^[22]^ The experimental group’s significantly improved theoretical examination scores (88.70 ± 5.07 vs 75.55 ± 9.01, *P* < .05) suggest that the NECP resource concentration and repeatability facilitate efficient knowledge acquisition. In contrast to the decentralized instruction of traditional training, theNECPprovides content recommendations tailored to trainees’ learning progress, such as analyses of common errors in cardiopulmonary resuscitation, enabling “precise remediation” and improving training outcomes.^[23]^ This personalized feedback mechanism enhances the acquisition of theoretical knowledge and operational skills, addressing issues related to skill-knowledge retention and review.^[24]^

The NECP operational video library provides standardized demonstrations for skills training, encompassing step-by-step breakdowns of emergency procedures (cardiopulmonary resuscitation) and injection methodologies (intramuscular injection). The experimental cohort’s enhanced operational proficiency scores (94.27 ± 2.04 vs 90.95 ± 3.69, *P* < .05) suggest that iterative video review and simulated practice effectively reduce operational ambiguities. Moreover, the NECP 3-station assessment module (emergency skills station, injection skills station, comprehensive skills station) enables trainees’ prompt refinement of operational nuances via immediate scoring and error feedback. This “learning-practicing-evaluating” closed-loop paradigm rectifies the delayed feedback characteristic of conventional training, consistent with the simulation training research of Wang^[25,26]^ and colleagues.

### 4.2. The strengthening of professional identity by the NECP

#### 4.2.1. Transparency and guidance in career development

The NECP assists nurses clarifying their career trajectories through the development of an electronic standardized-training manual and a career planning advice module. For instance, based on the assessment data and rotation performance of trainees, the system suggests specialized career paths in areas such as education and training, and intensive care.The increas in the total professional identity score of the experimental group (73.18 ± 10.18 vs 62.54 ± 15.48, *P* < .05) suggests that this personalized guidance enhances the sense of professional purpose of nurses.^[27]^ Furthermore, the platform features Nightingale Medal recipients sharing their professional experiences, thereby stimulating the professional sense of mission of trainees through role modeling, which alings with the perspective of CAO^[28,29]^ and others that “professional identity requires the integration of practice and spiritual guidance.” The NECP, by offering comprehensive learning resources and career development guidance, aids nurses in clarifying their career development paths. This support can improve the sense of control and belonging of standardized-training nurses regarding their careers, and subsequently strengthen their professional identity.^[30]^

#### 4.2.2. Real-time feedback and the enhancement of self-efficacy:

The NECP monitoring function provides real-time display of trainees’ learning progress and assessment rankings, prompting those who fall short of standards through early-warning notifications. This transparent feedback mechanism not only aids trainees in timely adjustments to their learning strategies but also bolsters self-confidence via positive reinforcement (e.g., the “Learning Star” list).^[31]^ Research indicates that the scores of the experimental group in the dimensions of “Professional Self-Concept” (25.63 ± 4.13 vs 22.14 ± 6.06, *P* < .05) and “Social Persuasion” (8.98 ± 1.34 vs 7.77 ± 2.28, *P* < .05) showed significant increases suggesting that the NECP has strengthened the professional value perception of nurses through a data-driven recognition mechanism.^[32,33]^ Furthermore, the systematic and continuous nature of training are also crucial. Following the implementation of personalized on-the-job training, the training satisfaction, professional quality, and work quality of standardized-training nurses all improved. Therefore, the NECP teaching model positively impacts training outcomes and can enhance teaching efficiency and quality.

### 4.3. The promoting effect of the NECP on clinical reasoning ability

#### 4.3.1. Ladder – type cultivation of clinical thinking:

The NECP divides clinical reasoning training into 3 stages: prejob stage: Cultivate basic reasoning ability through standardized case-analysis questions (such as the nursing plan for diabetic patients); Rotation stage: Require trainees to submit nursing problems of real cases on the platform and formulate solutions through literature retrieval and the guidance of teaching faculty; Fixed-department stage: Trainees need to complete evidence-based nursing projects (such as the pressure ulcer prevention strategy for ICU patients) and share the results across the department. The improvement in the clinical reasoning ability scores of the experimental group (60.95 ± 8.90 vs 51.09 ± 12.28, *P* < .05) indicates that this phased, task-driven training model effectively promotes the transformation of nurses from “memorizing knowledge to “applying knowledge.”^[34]^ The NECP achieves the deep integration of theory and practice through modular design.

#### 4.3.2. Support and promotion of evidence – based practice

The literature databases and evidence-based nursing tools (such as JBI evidence summaries) connected by the NECP provide a scientific basis for standardized-training nurses to solve clinical problems. For example, in the case discussion of “Infection Control in Sputum Suction Operations,” trainees can quickly retrieve the latest guidelines and optimize the operational process. This “problem-evidence-practice” closed-loop model not only improves the scientific nature of clinical decision-making but also cultivates the critical thinking skills of nurses.

### 4.4. The unique advantages of the NECP compared with traditional training

Traditional training is predicated on decentralized departmental instruction, which is susceptible to variations in instructor proficiency. Conversely, the NECP transcends the temporal and spatial constraints inherent in conventional training paradigms by consolidating high-quality resources across the entire hospital and facilitating fragmented learning, thereby augmenting learning flexibility. Its data-driven functionality enables real-time tracking of learning behaviors, precise delivery of personalized exercises, and a substantial enhancement in training efficacy. The phased design, progressing systematically from foundational to advanced levels, ensures training systematicity and coherence. This innovative modality not only mitigates the shortcomings of traditional training but also advances the standardization of nurse training toward precision and scientific rigor through technological integration.

### 4.5. Limitations and future research directions

#### 4.5.1. Research limitations

The study utilized a non-blinded, non-randomized design, which may have introduced measurement bias due to the evaluators’ awareness of group assignment. The absence of random assignment increased the risk of selection bias, potentially influenced by unobserved confounders such as learning motivation, technical adaptability, or historical effects (post-2021 hospital-wide training resource enhancements). To mitigate bias, standardized assessment tools were employed, including automated scoring for theoretical examinations, video-based skill scoring, and a dual-independent evaluator mechanism. PSM was also implemented to control for age, gender, and other baseline differences. Data analysis and results evaluation were performed independently by 2 researchers. Future investigations should consider a multicenter, randomized controlled design, incorporating third-party blinded assessments (qualitative interviews) to address confounding factors and investigate individual heterogeneity in intervention outcomes.^[35]^

Single-center sample limitation: The data of this study were exclusively sourced from a top-3 hospital in Chongqing. The homogeneity of the sample (hospital grade, regional economic level) may affect the generalizability of the conclusions. For instance, nurses in primary-level hospitals or economically underdeveloped regions may encounter issues such as resource scarcity and inadequate network coverage, potentially limiting the application effect of the NECP. Similar studies have indicated that multicenter data can mitigate the influence of regional and institutional variations on the results.^[36]^ Future research should incorporate samples from diverse-level medical institutions (secondary-level hospitals, community hospitals) and multiple regions to validate the broad applicability of the model.

Technical dependence risks: the NECP functionality is contingent upon the network infrastructure and digital devices. Certain nurses or trainees with limited technological proficiency may experience compromised learning outcomes due to operational challenges.Prior research indicates that trainees require supplementary technical support during their initial engagement with online learning platforms.^[37]^ It is recommended to add a “Newcomer Guidance Module” (operational videos, real-time online customer service) into the platform design and offer supplementary offline technical training ssnoisses to mitigate the impact of the digital divide on the training efficacy.

Insufficient long-term effect tracking: This study only evaluated the short-erm effects following the conclusion of the training and lacks long-term tracking of the career development of standardized-training nurses (job competency after 3–5 years). The literature indicates that the sustainability of the training effect necessitates joint evaluation in conjunction with on-the-job practice and continuing education.^[38]^ Future research should establish a longitudinal research cohort can be established to analyze the long-term impact of the NECP training on indicators such as the career advancement and turnover rates of standardized-training nurses.

Uncontrolled confounding variables: despite the absence of significant differences in the baseline data between the 2 groups, potential confounding factors (the personality traits and self-learning abilities of trainees) were not controlled. For instance, nurses with high self-efficacy may exhibit greater proactivity in utilizing the NECP resources, thereby amplifying the experimental group’s advantages.^[39]^ It is recommended that future research employ a mixed-method approach (quantitative data combined with qualitative interviews) to analyze the heterogeneity of the training effect, considering trainees’ characteristics.

#### 4.5.2. Future improvement directions

Expansion of Intelligent Functions:Implement artificial intelligence-assisted assessment technologies (using voice recognition technology to analyze the nurse-patient communication ability) to further improve the real-time and accuracy of feedback.Simultaneously, an intelligent dialogue system founded on natural language processing can be developed to simulate authentic clinical dialogue scenarios, thereby assisting standardized-training nurses improve their communication skills and response abilities in practice.Furthermore, through deep-learning algorithms, analyze the performance of trainees in simulated dialogs, identify communication barriers, and provide targeted improvement recommendations, thereby comprehensively augmenting the comprehensive abilities of standardized-training nurses.

Development of multimodal learning resources: integrate virtual reality technology to simulate complex clinical scenarios such as emergency rescue to enhance the effect of contextualized training. Simultaneously, the integration of augmented reality technology into training can be explored. By superimposing virtual information on the real environment in real-time, standardized-training nurses can obtain a more intuitive and immersive learning experience in simulated operations. For instance, in intravenous infusion procedures, trainees can more accurately master the skills of venipunctureg and reduce errors in actual operations. Furthermore, mobile learning applications can be developed to utilize fragmented time for knowledge-point review and skill-simulation exercises, thereby further improving learning efficiency.

## 5. Conclusions

Through resource integration, data-driven design, and phased implementation, the NECP has demonstrably enhanced the efficacy of standardized nurse training. Initially, the platform integrates standardized resources with a personalized feedback mechanism, thereby facilitating nurses’ efficient acquisition of knowledge and skills, and accelerating the translation of theoretical concepts into practical application. Secondly, transparent career pathway planning and a real-time incentive structure have augmented nurses’ professional identity and sense of affiliation. Finally, the task-driven, evidence-based practice model has systematically cultivated nurses’ clinical reasoning abilities and fostered the development of scientific decision-making capabilities. Future investigations may further explore the integration of the NECP with emerging technologies to construct a more intelligent, precise, and clinically-relevant nurse training paradigm.

## Acknowledgments

The authors thank all the participants and research assistants involved in this study. Additionally, all authors extend their gratitude to the standardized-training nurses who generously devoted their time to participate in this research.

## Author contributions

**Conceptualization:** Haoyu Tang, Huaxi Liu.

**Data curation:** Yuan Yuan.

**Formal analysis:** Shaoyi Hu.

**Investigation:** Huaxi Liu.

**Methodology:** Shaoyi Hu.

**Resources:** Shaoyi Hu.

**Software:** Yuan Yuan.

**Supervision:** Shaoyi Hu.

**Validation:** Haoyu Tang, Yuan Yuan, Huaxi Liu.

**Visualization:** Haoyu Tang.

**Writing – original draft:** Haoyu Tang.

**Writing – review & editing:** Shaoyi Hu.
